# Screening of Dengue Virus Antiviral Activity of Marine Seaweeds by an *In Situ* Enzyme-Linked Immunosorbent Assay

**DOI:** 10.1371/journal.pone.0051089

**Published:** 2012-12-05

**Authors:** Andrea Cristine Koishi, Paula Rodrigues Zanello, Éverson Miguel Bianco, Juliano Bordignon, Claudia Nunes Duarte dos Santos

**Affiliations:** 1 Programa de Pós-graduação em Biologia Celular e Molecular, Universidade Federal do Paraná (UFPR), Curitiba, Paraná, Brazil; 2 Laboratório de Virologia Molecular, Instituto Carlos Chagas (ICC–FIOCRUZ/PR), Curitiba, Paraná, Brazil; 3 Programa de Pós-graduação em Química, Universidade Federal de Pernambuco (UFPE), Recife, Pernambuco, Brazil; CSIR-Institute of Microbial Technology, India

## Abstract

Dengue is a significant public health problem worldwide. Despite the important social and clinical impact, there is no vaccine or specific antiviral therapy for prevention and treatment of dengue virus (DENV) infection. Considering the above, drug discovery research for dengue is of utmost importance; in addition natural marine products provide diverse and novel chemical structures with potent biological activities that must be evaluated. In this study we propose a target-free approach for dengue drug discovery based on a novel, rapid, and economic *in situ* enzyme-linked immunosorbent assay and the screening of a panel of marine seaweed extracts. The *in situ* ELISA was standardized and validated for Huh7.5 cell line infected with all four serotypes of DENV, among them clinical isolates and a laboratory strain. Statistical analysis showed an average S/B of 7.2 and Z-factor of 0.62, demonstrating assay consistency and reliability. A panel of fifteen seaweed extracts was then screened at the maximum non-toxic dose previously determined by the MTT and Neutral Red cytotoxic assays. Eight seaweed extracts were able to reduce DENV infection of at least one serotype tested. Four extracts (Phaeophyta: *Canistrocarpus cervicornis*, *Padina gymnospora*; Rhodophyta: *Palisada perforate*; Chlorophyta: *Caulerpa racemosa*) were chosen for further evaluation, and time of addition studies point that they might act at an early stage of the viral infection cycle, such as binding or internalization.

## Introduction

Dengue is the most important mosquito-borne viral disease in the world. It is a significant public health concern that transcends geographical boundaries, being endemic in more than 100 countries within tropical and subtropical regions of the world [1]. It is believed that two-fifth of the world’s population live in infection-risk areas and that around 50 million new infections and 25 thousand deaths occur annually [1], [2]. The total economic burden of dengue illness in the Americas was estimated to cost US$ 2.1 billion per year on average [3]. However, this value is most likely underestimated due to a large number of non-reported cases.

Dengue virus (DENV) belongs to the *Flavivirus* genus in the *Flaviviridae* family, and is presently classified into four different serotypes (DENV-1, -2, -3 and -4), and all of them are capable of causing the disease. Mature virions present a positive single-stranded RNA genome enclosed by a nucleocapsid exhibiting icosahedral symmetry, with the envelope and membrane proteins protruding from the host lipid bi-layer membrane [4]. The viral genome of approximately 11 Kb presents a Cap at its 5′ end and has a single open reading frame that encodes for one polyprotein that is cleaved in the course of and post translation in 3 structural and 7 non-structural proteins (5′-C-prM(M)-E-NS1-NS2A-NS2B-NS3-NS4A-NS4B-NS5-3′) [5].

Clinical manifestations of DENV infection vary from an undifferentiated fever (dengue fever, DF) to more severe forms like dengue hemorrhagic fever (DHF) and dengue shock syndrome (DSS), which can lead to death. The severity of disease might be related to strain virulence, host factors and/or secondary infections with heterologous serotypes [6]–[8] suggesting that immunopathological mechanisms are involved with the disease progression [9], [10].

**Figure 1:**
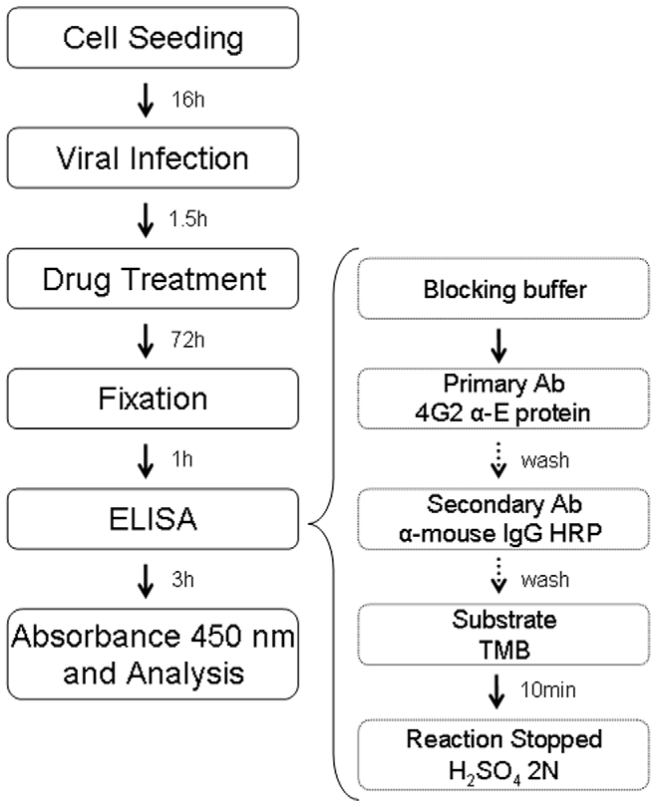
*In situ* ELISA assay workflow.

Despite the important social and clinical impact of the disease, dengue treatment is palliative. At present, there is no FDA-approved vaccine or specific antiviral therapy for prevention and treatment of dengue. Considering the described scenario, drug discovery research for dengue is of great importance. To our knowledge, there are currently ten drug discovery programs in the pre-clinical or discovery stages for dengue, including RNAi therapeutics and natural products. However there are still no antiviral drugs being tested against dengue disease in any clinical trial [11].

Some reports demonstrate possible dengue viral inhibitors to different targets as viral adsorption and entry [12], NS3 protein [13], RNA replication and viral translation [14] or host processes [15]. Yin *et al*. (2009) [16] characterized an adenosine analog capable of reduce viremia, TNF-α and IL-6 production, preventing the death of infected mouse. Among the compounds tested, there are thousands of synthetic small molecules and natural products. Recently, the study of the biological activity of seaweeds extracts became a growing field of interest with the isolation and characterization of thousands of novel compounds with pharmacological properties from different marine organisms [17].

The antiviral activity screening of compounds is accomplished using several approaches, a number of them are low-throughput as the plaque reduction assay that is laborious, time consuming and have a subjective measurement. On the other hand, there are a number of high-throughput assays reported for anti-dengue infection screening [18]–[20], which are quantitative and can test a large number of compounds, however they often use new technologies as equipments, robots and software that are expensive and of restrict use.

To circumvent these issues, some researchers used an *in situ* ELISA for the screening of antiviral agents for influenza A virus [21]; varicella-zoster virus [22] and human cytomegalovirus [23]. Based on that, we propose a simple target-free approach for dengue drug discovery using a cell based ELISA, which is adaptable to automation and provides objective and rapid results, making use of materials and reagents common to many laboratories. The assay was standardized, validated and used to screen a panel of chemical compounds present in seaweed extracts.

**Table 1:** Marine seaweeds selected for antiviral screening, location of collection and cytotoxicity activity in Huh7.5 cells.

Seaweed Species	Location (Latitude/Longitude)	CC50 (µg/ml)^a^			MNTD^b^(µg/ml)	Extract Code
		Neutral Red	MTT			
**Phylum Phaeophyta** c						
*Canistrocarpus cervicornis* (Kützing)De Paula & De Clerck	Paraíso Beach (08°21’S; 34°57’W)	127.0		86.56	31.25	A1
*Dictyota mertensii* (Martius) Kutzing	Itapuama Beach (08°17’S; 34°57’W)	111.7		77.07	31.25	A15
*Dictyopteris delicatula* J.V. Lamour	Calhetas Beach (08°20’S; 34°56’W)	>250		>250	125	A2
*Padina gymnospora* (Kütz.) Sond.	Boa Viagem Beach (08°07’S;34°53’W)	>250		>250	125	A3
*Lobophora variegata* (J. V. Lamour.)Womersley ex E. C. Oliveira	Calhetas Beach (08°20’S; 34°56’W)	>250		148.5	62.5	A4
*Sagassum vulgare* var. *nanun* E. de Paula& E. C. Oliveira	Paraíso Beach (08°21’S; 34°57’W)	237.7		217.3	62.5	A5
*Sargassum vulgare* var. *vulgari* C. Agardh	Paraíso Beach (08°21’S; 34°57’W)	224.1		233.2	62.5	A6
**Phylum Rhodophyta** c						
*Digenia simplex* (Wulfen) C. Agardh	Suape Beach (08°22’S; 34°56’W)	>250		85.26	62.5	A9
*Laurencia dendroidea* J. Agardh	Suape Beach (08°22’S; 34°56’W)	1.069		0.6260	<0.5	A7
*Palisada perforata* (Bory) K.W. Nam	Suape Beach (08°22’S; 34°56’W)	>250		239.5	125	A8
*Hypnea musciformis* (Wulfen in Jacquin)J. V. Lamour.	Paraíso Beach (08°21’S; 34°57’W)	>250		101.6	31.25	A10
*Gracilaria* sp.	Viagem Beach (08°07’S; 34°53’W)	>250		>250	125	A11
**Phylum Chlorophyta** c						
*Chaetomorpha antennina* (Bory) Kütz.	Suape Beach (08°22’S; 34°56’W)	91.40		84.38	31.25	A13
*Dictyosphaeria cavernosa* (Forssk.)	Suape Beach (08°22’S; 34°56’W)	79.13		66.05	31.25	A14
*Caulerpa racemosa* (Forssk.) J. Agardhvar. *racemosa*	Suape Beach (08°22’S; 34°56’W)	178.6		121.8	62.5	A12

a CC50∶50% cytotoxic concentration (µg/ml).

b MNTD: maximum non- toxic dose based on MTT and Neutral Red assays.

c Phaeophyta = Brown seaweeds; Rhodophyta = Red seaweeds; Chlorophyta = Green seaweeds.

## Materials and Methods

### Cell Lines and Viruses

Human-derived hepatoma cells (Huh7.5, ATCC PTA-8561) were grown in Dulbecco’s Modified Eagle Medium: Nutrient Mixture F-12 (D-MEM/F-12 medium) (Gibco/Invitrogen, Grand Island, NY, USA) supplemented with 10% fetal bovine serum (FBS) (Gibco/Invitrogen, Grand Island, NY, USA) and 100 IU/µg/ml penicillin/streptomycin (Gibco/Invitrogen, Grand Island, NY, USA) at 37°C in an humidified, 5% CO_2_-controlled atmosphere. C6/36 *Aedes albopictus* cells (ATCC CRL-1660) were grown in Leibovitz L-15 medium (Gibco/Invitrogen, Grand Island, NY, USA) supplemented with 5% FBS, 0.26% tryptose (Sigma-Aldrich, St. Louis, MO, USA) and 25 µg/mL gentamicin (Gibco/Invitrogen, Grand Island, NY, USA) at 28°C.

**Figure 2:**
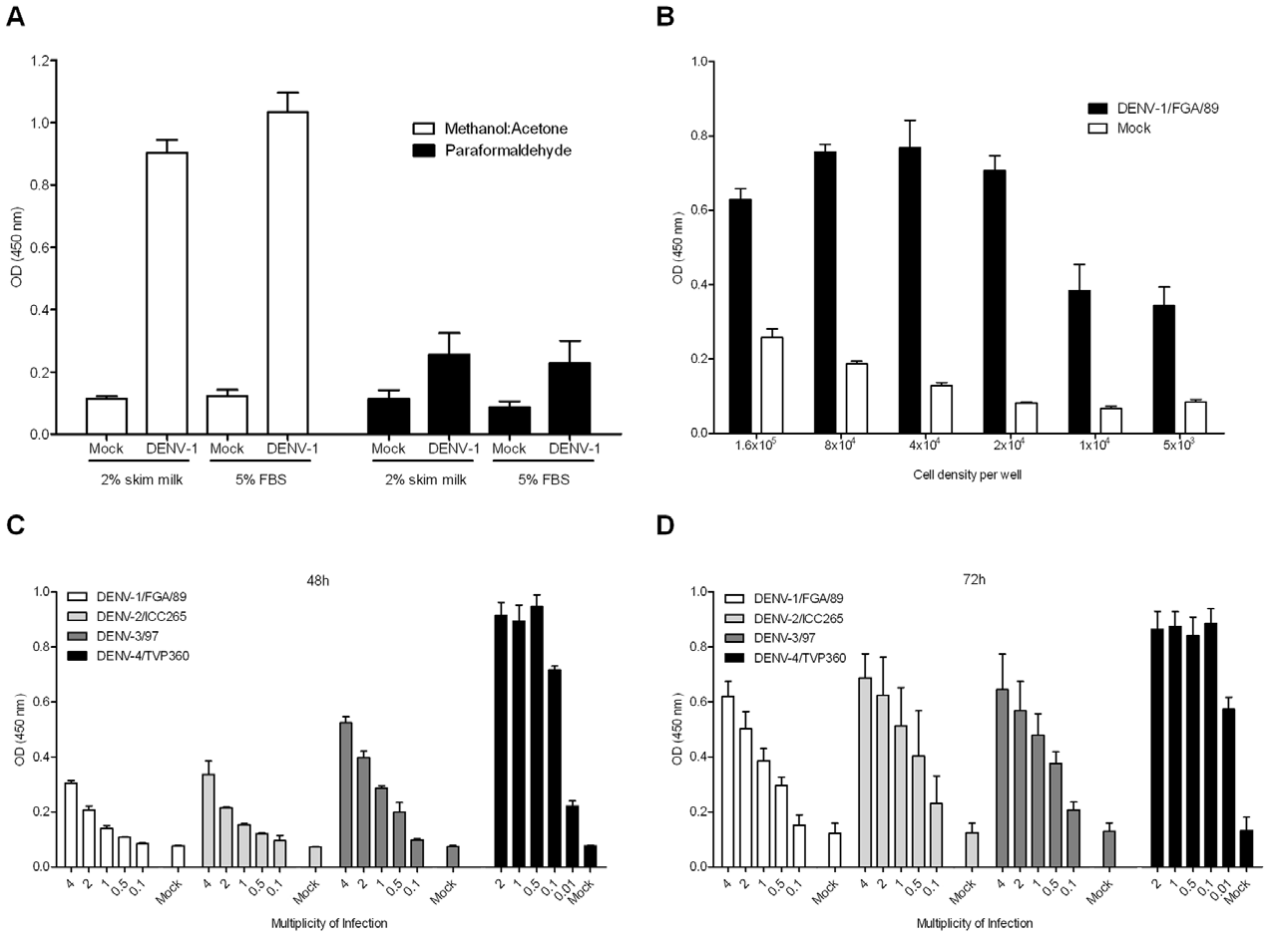
*In situ* ELISA optimization. (**A**) Effect of different blocking buffers (skim milk and FBS) and fixatives (methanol:acetone and paraformaldehyde followed by triton X-100) for the assay. (**B**) Cells were seeded in a range of concentration and the influence for the assay performance was evaluated. (**C**) The ideal MOI for all DENV serotypes was evaluated 48 hours post-infection and (**D**) 72 hours post-infection. Values represent mean ± SD of three independent experiments performed in triplicate.

DENV-1/FGA/89 was isolated from a patient with dengue fever in South America in 1989 and kindly supplied by Dr. Philippe Desprès, from Unite des Interactions Moléculares Flavivurs-Hôtes from Pasteur Institut, Paris, France. DENV-1/BR/90, DENV-2/BR/01-01, DENV-2/ICC265 and DENV-3/97 are clinical isolates from dengue fever obtained in Brazil between the years of 1990 and 2004. DENV-3/5532 was isolated from a fatal case of dengue with visceral complications in Lambaré, Paraguay in 2007. DENV-4/TVP360 is a laboratory strain kindly supplied by Dr. Ricardo Galler from Fundação Oswaldo Cruz, Rio de Janeiro, Brazil. Virus stocks were propagated in C6/36 cells and titrated by foci-forming immunodetection assay.

**Figure 3:**
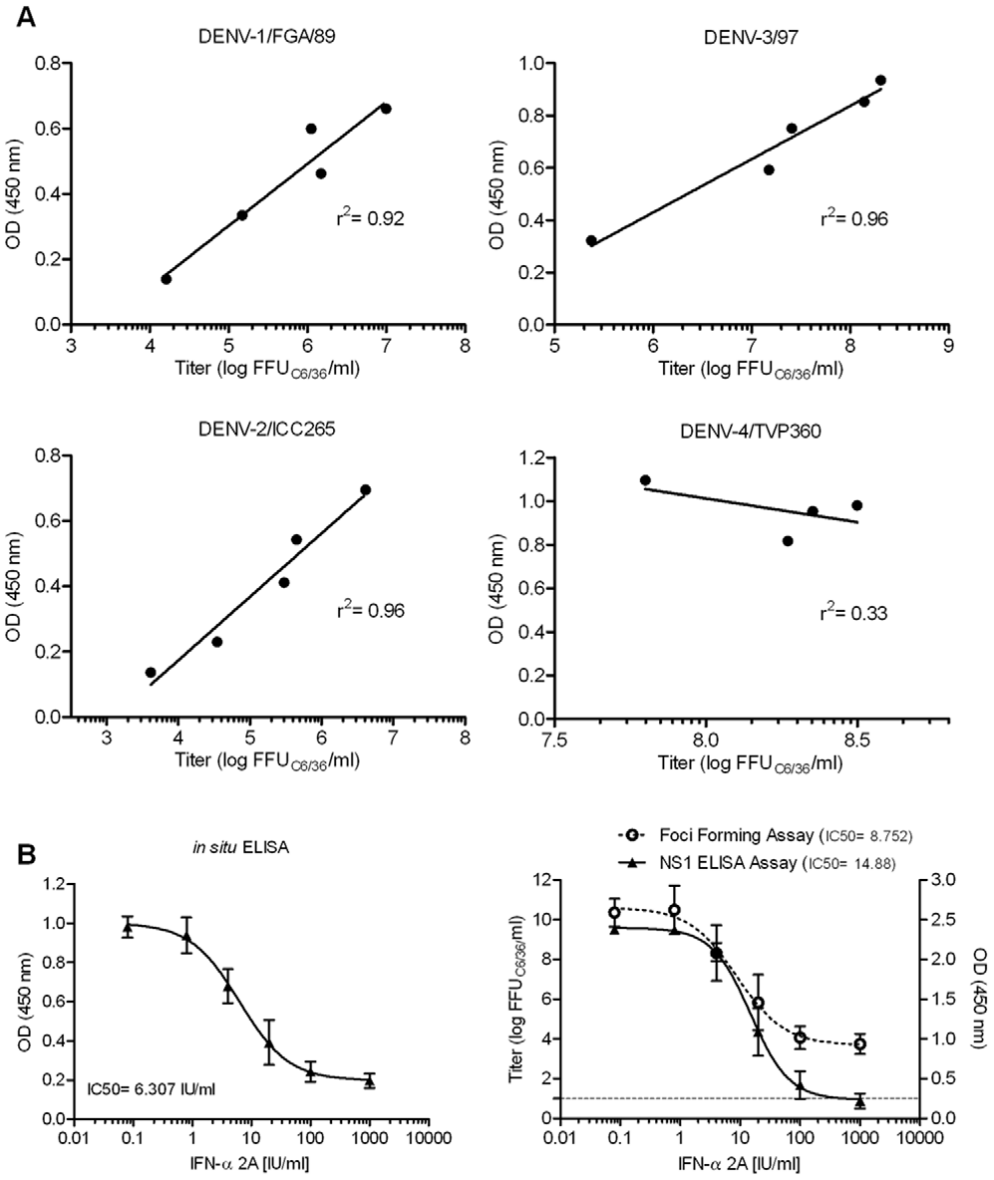
*In situ* ELISA assay validation. (**A**) Correlation between the *in situ* ELISA and the foci-forming assay, where Huh7.5 was infected with DENV-1, DENV-2, DENV-3 and DENV-4 at a range of MOI (4–0.01). After 72 hours of infection the supernatant was used for the foci-forming assay and the cells submitted to the ELISA assay. Data from one representative experiment, analyzed using Pearson correlation test. (**B**) Comparison of the DRC and the IC50 for IFN-α 2A and DENV-4 infection, obtained with the *in situ* ELISA, foci-forming assay and a commercial NS1 antigen capture ELISA assay. Mean ± SD of three independent experiments, analyzed by sigmoidal dose-response curve (variable slope), the dashed line represents the detection limit of the foci-forming assay.

### Foci-forming Immunodetection Assay

Viral titers were determined by the foci-forming immunodetection assay in C6/36 cells (FFU_C6/36_), as previously described [24]. Briefly, cell culture supernatants were serially diluted and added to 24 well titration plates (TPP, Trasadingen, Switzerland) in duplicate. After 1 h30 min, the inoculum was removed and a CMC overlay media (L-15 supplemented with 10% SFB, 0.52% tryptose, 50 µg/mL gentamicin, 1.6% carboxymethylcellulose) was added. The immunostaining was performed after seven days using the mouse monoclonal *Flavivirus* group-specific antibody 4G2 (hybridoma D1-4G2-4-15, ATCC HB-112), followed by goat anti-mouse immunoglobulin conjugated to alkaline phosphatase (Promega, Madison, WI, USA), which was detected by adding a solution of NBT (nitroblue tetrazolium chloride) and BCIP (5-bromo-4-chloro-39-indolyphosphate p-toluidine salt) (Promega, Madison, WI, USA) as a substrate. Foci was counted and expressed as FFU_C6/36_/ml.

**Figure 4:**
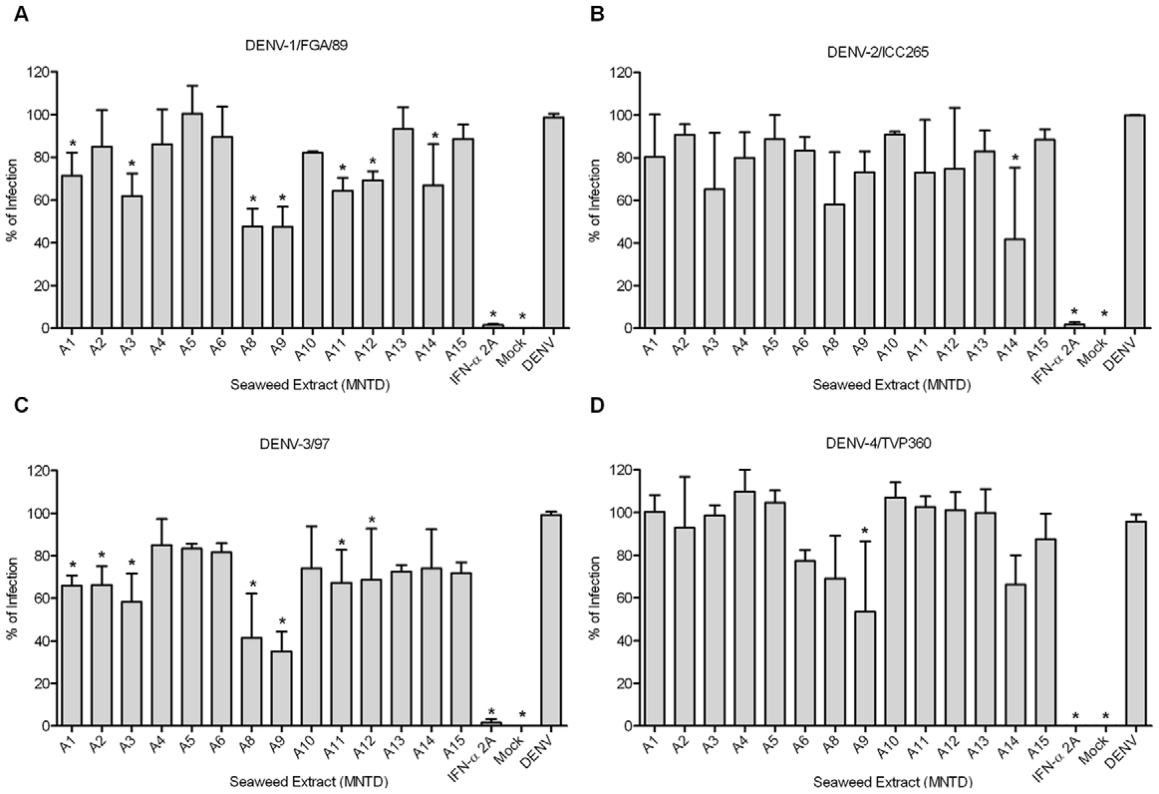
Seaweed extracts antiviral screening. (**A**) Huh7.5 were seeded in a 96-well plate and infected with DENV-1, (**B**) DENV-2 and (**C**) DENV-3 with an MOI of 4, and (**D**) DENV-4 with an MOI of 0.1. Interferon-α 2A (100 IU/ml) was used as a positive control and after 72 hours post-infection the ELISA was performed. Data were analyzed using one-way ANOVA followed by Tukey test. Values are mean ± SD of three independent experiments. *p<0.05.

### *In situ* Cellular Enzyme-linked Immunosorbent Assay Optimization and Validation

Several parameters as optimal cell density per well (1.6×10^5^–5×10^3^), multiplicity of infection (MOI of 4–0.01) for each virus serotype, cell fixation reagents (methanol:acetone or paraformaldehyde followed by permeabilization with triton X-100), blocking buffers (FBS or skim milk) and time of incubation after infection (48 h or 72 h) were analyzed.

**Figure 5:**
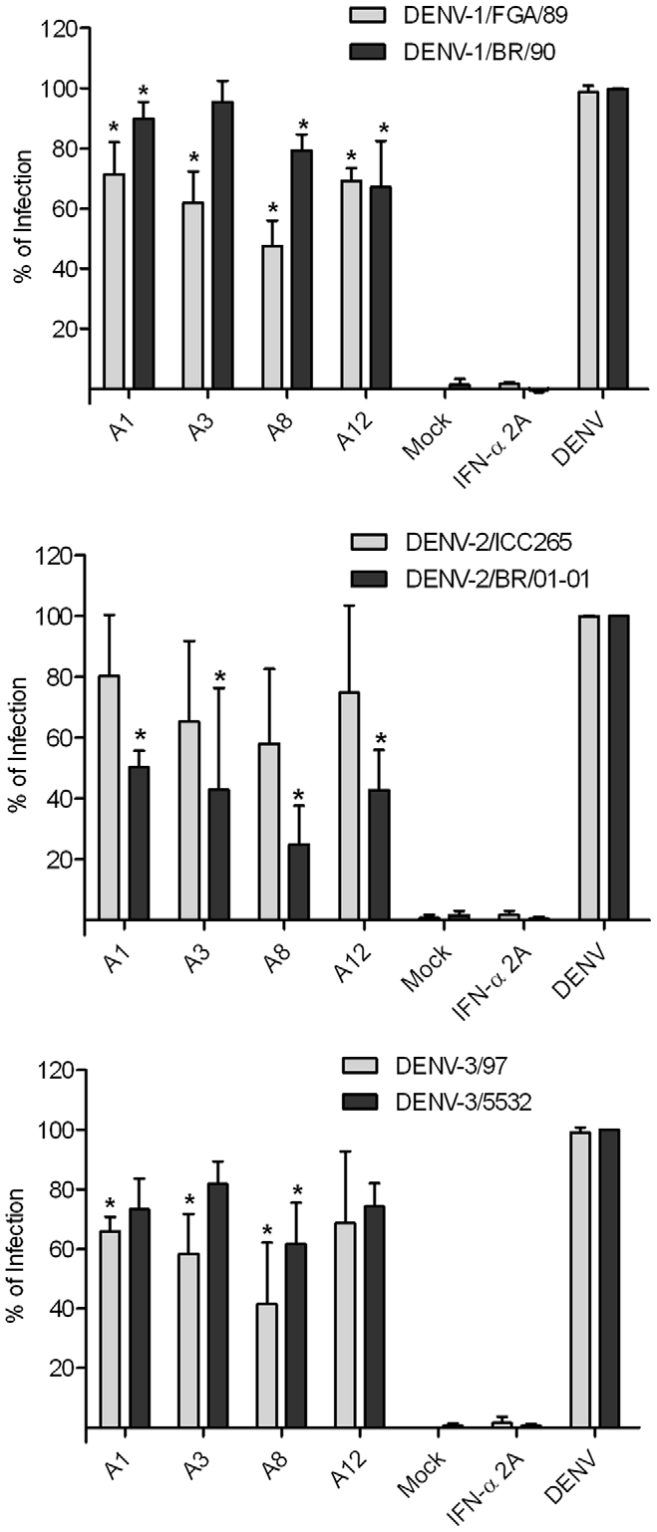
DENV infection inhibition for different strains/serotypes. Huh7.5 was seeded in a 96-well plate and infected with each strain with a MOI of 4 for 1 h30 min, then the seaweed extracts were added and after 72 hours the ELISA was performed. Data were analyzed using one-way ANOVA followed by Dunnett test. Values are mean ± SD of three independent experiments,*p<0.05.

The best conditions were determined and used for the subsequent tests. Briefly, after fixation with methanol:acetone, blocking buffer (2% skim milk, 0.05% Tween-20 PBS) was added and after 30 min the primary antibody 4G2 was added. Plates were incubated for 1 h at 37°C and then washed four times with washing buffer (0.01% Tween-20 PBS). The secondary antibody goat anti-mouse IgG HRP (Sigma-Aldrich, St. Louis, MO, USA) was added and after 1 h incubation at 37°C, plates were washed four times with washing buffer. TMB substrate (KPL, Gaithersburg, USA) was added and incubated for 10 min to allow color development; the reaction was stopped with 2N H_2_SO_4._ Absorbance was read at a wavelength of 450 nm in a microplate reader (Synergy H1 M, Biotek, USA) (Figure 1).

**Figure 6:**
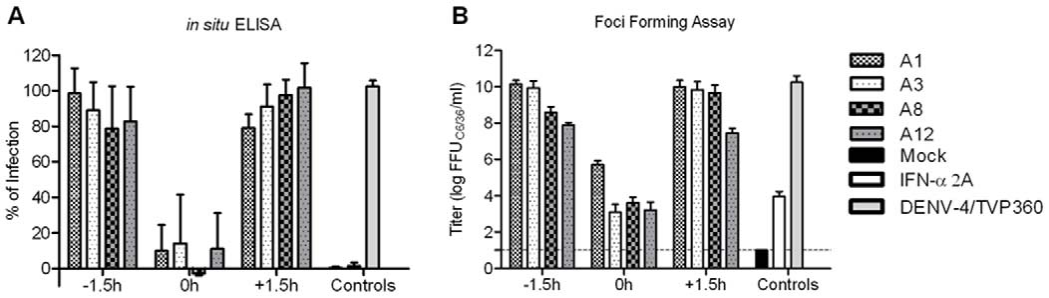
Time of addition studies with seaweed extracts A1, A3, A8 and A12. (**A**) Huh 7.5 cells were infected with DENV-4 with an MOI of 0.1 following each treatment (before the infection, −1.5 h; during the infection, 0 h; and after the infection, +1.5 h) at the MNTD, the cells were submitted to the ELISA assay (**B**) and the supernatant for the foci-forming assay. The dashed line represents the detection limit of the foci-forming assay.

For assay validation, Huh7.5 cells were infected with all DENV serotypes at a range of MOIs and after 72 h the *in situ* ELISA and the foci-forming immunodetection assay from the same culture were compared by way of Pearson’s correlation coefficient. A second validation was done by infecting Huh7.5 cells with DENV-4 at a MOI of 0.1, followed by treatment with IFN-α 2A (Blausiegel) in a dose response curve. The results of the *in situ* ELISA, the foci-forming assay and a commercial NS1 antigen capture ELISA (Panbio Dengue Early ELISA - second generation, Alere, Australia) were compared. Interferon-α 2A was then used as a reference control and the IC50 (concentration that inhibits 50% of virus infection) was determined using a DRC for all dengue virus serotypes.

**Figure 7:**
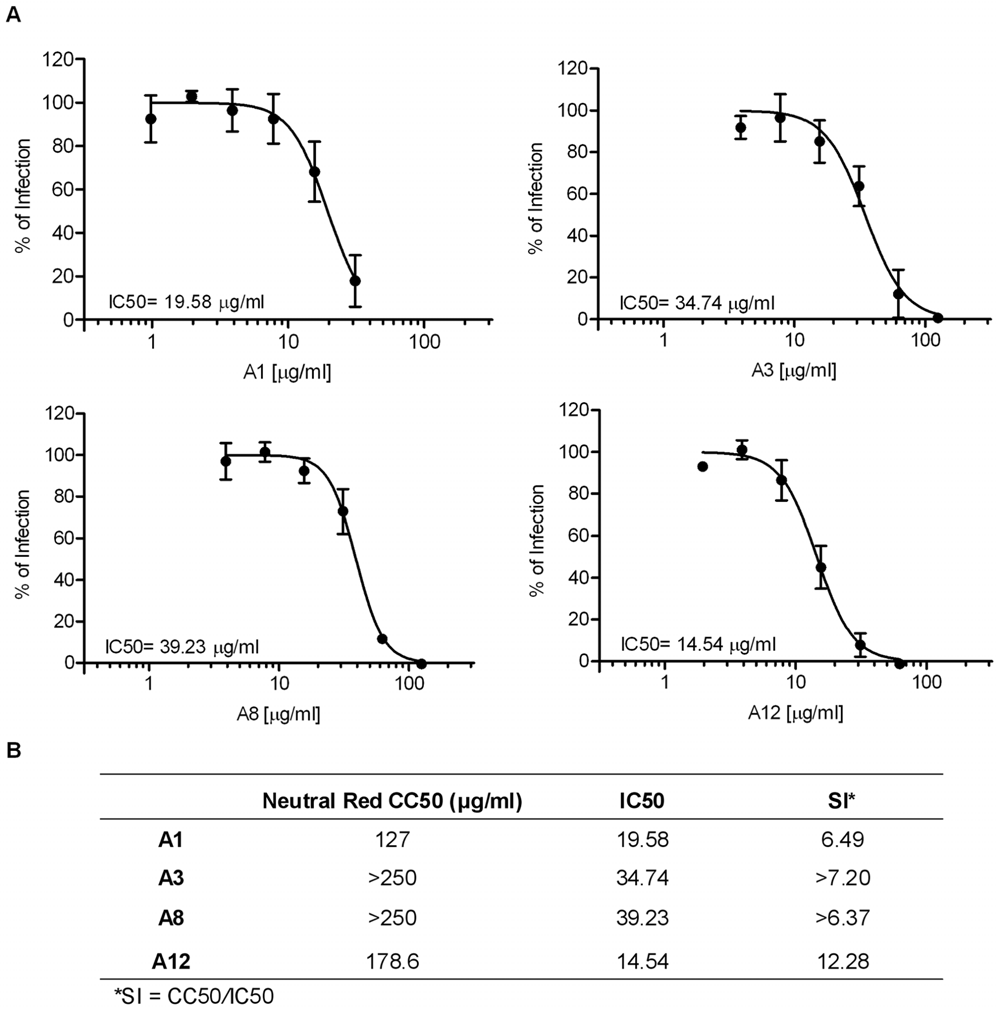
Dose response curve for the seaweed extracts A1, A3, A8 and A12. (**A**) Huh 7.5 cells were infected with DENV-4 and treated during the infection in a range of concentration for each seaweed extract. (**B**) CC50, IC50 and SI were calculated. Mean ± SD of three independent experiments, analyzed by sigmoidal dose response curve (variable slope).

### Seaweed Collection

Marine seaweeds from the phylum Phaeophyta, Rhodophyta, and Chlorophyta were collected in the intertidal zone of Pernambuco State coast, Brazil, in August-October 2009 (Table 1). The seaweed material was cleaned manually from epiphytic organisms immediately after collection, and air-dried. Specimens were identified and voucher specimens were deposited at the Botany Herbarium of the Biology Department at Pernambuco Federal University, Brazil.

**Figure 8:**
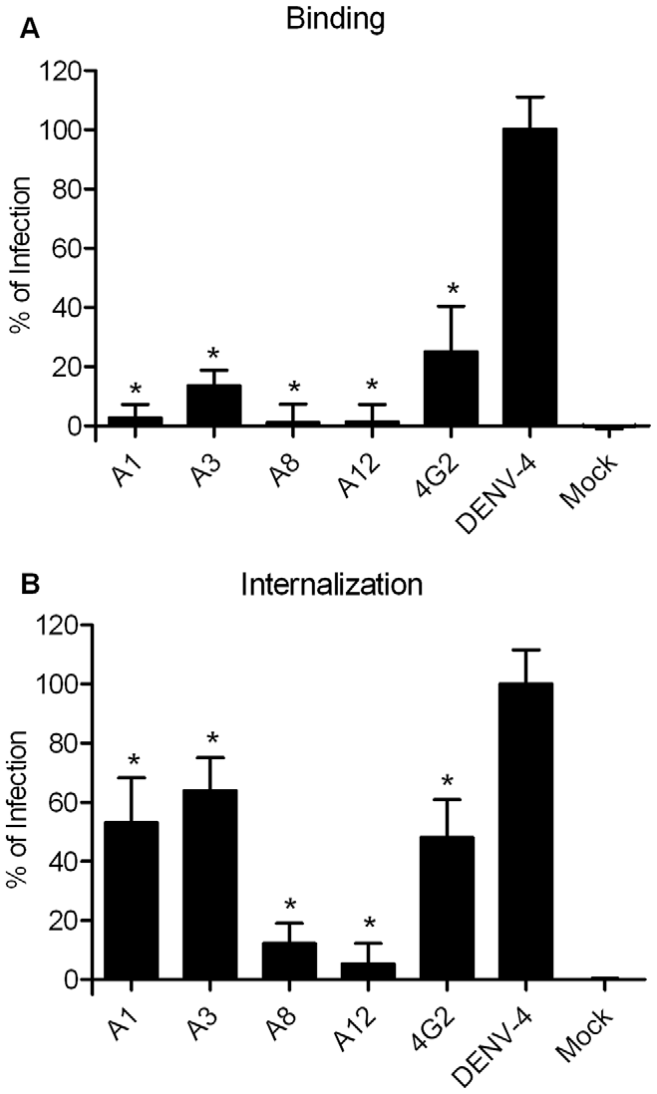
Effect of the seaweed extracts A1, A3, A8 and A12 on DENV-4 entry process. (**A**) Binding inhibition assay. (**B**) Internalization inhibition assay. Data were analyzed using one-way ANOVA followed by Dunnett test. Values are mean ± SD of three independent experiments,*p<0.05.

### Chemical Extraction of Marine Seaweeds

Air-dried algal material (10 g per species) were exhaustively extracted at room temperature with dichloromethane/methanol (2∶1), yielding 15 dichloromethane/methanol crude extracts, which were evaporated to dryness at low temperature (<50°C) on a rotary evaporator [25], which was resuspended in 100% dimethyl sulfoxide (DMSO) for a concentration of 25 mg/ml and stored in aliquots protected from the light at −20°C.

### Cell Viability Assay

Seaweed extracts cytotoxicity was assessed simultaneously by the MTT [3-(4,5-dimethylthiazol-2-yl)-2,5-diphenyl tetrazolium bromide] and Neutral Red (N-Red) assays [26]. Briefly, Huh7.5 cells were cultivated into a 96 well flat bottom plate at a density of 2×10^4^ cells/well. After 16 hours of incubation cells were treated with the seaweed extracts in a two fold serial dilution in triplicate and incubated at 37°C and 5% CO_2_. A blank control (medium only) and cell control (cells only) were also included in every assay plate. After 72 h of incubation, the supernatant was removed and 33 µg/ml of N-Red reagent was added to each well and the plate was incubated for 1 h at 37°C, 5% CO_2_. Additionally, 0.5 mg/ml of MTT was added for another 2 h at 37°C and 5% CO_2_. After removal of the medium, 150 µl of extracting solution (1% acetic acid, 30% ethanol) was added to each well and incubated for 15 min under agitation to extract the N-Red dye. The solution was then transferred to another microplate, and the absorbance was determined by spectrophotometry at 550 nm. The MTT formazan crystals were extracted by adding 150 µl of DMSO. Optical density was measured at 550 nm and 630 nm differential reading with a microplate reader (Synergy H1 M, Biotek, USA). Data were normalized following the equation: Cell viability (%) = (sample value ­ blank control)/(cell control - blank control)× 100. A dose response curve was obtained using a non-linear regression (curve fit), and the cytotoxic concentration 50% (CC50) was calculated as the concentration required to reduce cell viability by 50%.

### Antiviral Screen of Marine Seaweed Extracts

The seaweed extracts were screened in triplicate at the maximum non-toxic dose (MNTD) in 1% DMSO, which was determined by the MTT and N-Red assays. A negative control (1% DMSO medium) and a positive control (EC90 for Interferon-α 2A) were included in all test plates. Huh7.5 cells were seeded at a density of 2×10^4^ cells per well on a flat-bottom 96-well plate, 16 h prior to infection. Culture medium was removed and 100 µl of viral inoculum (MOI of 4 for DENV-1, -2 and -3; and MOI of 0.1 for DENV-4) was added to wells and plates were incubated for 1 h30 min at 37°C, 5% CO_2_. The inoculum was replaced with 200 µl of medium or each seaweed extract dilution. After a 72 h incubation period, cells were fixed and the ELISA performed as previously described. Data were normalized as % of infection in relation to the controls, where the OD obtained with the non-infected culture were taken as 0% infection, and the one obtained with the non-treated infected culture as 100% infection.

### Time of Addition Assays

Huh7.5 cells at a density of 2×10^4^ cells per well were infected with DENV-4 at a MOI of 0.1 and the viral antigen quantification was performed by the *in situ* ELISA and viral progeny by the foci-forming assay. In this experiment, three different time points were tested. The MNTD of the seaweed extracts A1, A3, A8 and A12 were added 1 h30 min before the infection (i), during the infection (time 0 h) (ii) and 1 h30 min after the infection (iii). Furthermore, the during infection treatment was performed with a serial dilution of each seaweed extract, and a DRC, IC50 and the selectivity index (SI =  CC50/IC50) were calculated.

### Binding and Internalization Assays

The assays were performed as previously described [27] with some modification. Briefly, the virus binding assay was done with Huh7.5 cells seeded in 96-well plates and infected with DENV-4 with a MOI of 0.1 in the presence or absence of the seaweed extracts at the MNTD and incubated for 1 h at 4°C. The inoculum was removed and the cells washed twice with cold PBS. Complete medium was added to wells and the plates were incubated at 37°C, 5% CO_2_ for 72 h. After the incubation period the *in situ* ELISA was performed as described before.

For the virus internalization assay, Huh7.5 cells were seeded in 96-well plates and infected with DENV-4 with a MOI of 0.1. After an incubation of 1 h at 4°C, the non adsorbed virus was removed and cells were washed with PBS. Medium with or without the seaweed extracts at the MNTD were added to wells and incubated at 37°C for 1 h. After the post-adsorption period, cells were washed with PBS and treated with 0.1 ml of citrate buffer (citric acid 40 mM, potassium chloride 10 mM, sodium chloride 135 mM, pH 3) for 1 min to inactivate adsorbed but not internalized virus. Cells were then washed with PBS and complete medium was added, plates were then incubated at 37°C, 5% CO_2_ for 72 h and the ELISA was performed as previously described. For both assays the neutralizing antibody 4G2 was used as a control.

### Virucidal Assay

The virucidal assay was done as described by Burghgraeve *et al*. (2012) [28] with some modifications. DENV-4 (2×10^5^ FFU_C6/36_/ml) was incubated at 37°C in the presence or absence of the seaweed extracts at the MNTD and the positive control consisted of purified virus RNA. All samples were incubated in the presence or absence of 150 µg/ml RNase A (USB). After one hour, viral RNA was isolated using QIAamp Viral RNA Mini Kit (QIAGEN) and the samples were subjected to RT-PCR [29] and gel electrophoresis.

### Statistical Analysis

After normalization, data were analyzed using one-way ANOVA followed by Tukey or Dunnett tests; Pearson correlation test was used to compare different assays; and the sigmoidal dose-response (variable slope) for CC50 and IC50. The level of significance for the analysis was set at p<0.05. The analysis were performed with Prism Software (GraphPad version 5.0c, San Diego, CA, USA).

Assay performance was analyzed by measurements of the signal to background S/B = (M_max_/M_min_), and the Z-factor = 1– [3× (SD_max_+SD_min_)/(M_max_− M_min_)] where M_max_ and M_min_ represent the mean optical density values of positive and negative controls, and SD_max_ and SD_min_ are the respective standard deviations for those [30].

## Results and Discussion

### Optimization of the *in situ* ELISA Assay

Despite being the most important mosquito-borne viral disease in the world, currently there is no specific antiviral therapy available for dengue infection treatment. A safe and efficient anti-dengue virus drug should ideally have the potential to reduce the total number of people developing clinical manifestations of the disease and provide protection for people who travel to regions where DENV is endemic.

To accomplish this, there are a number of requirements to be fulfilled. An anti-dengue drug should have an easy route of administration, stability, a long shelf-life and be reasonably priced. With respect to clinical efficacy, the drug must be active against all four dengue serotypes in both primary and secondary DENV infections, with a good safety profile [1].

Therefore the development of DENV specific antiviral drugs is of high priority to improve the disease scenario worldwide. To identify inhibitors of DENV infection, a 96-well format cell-based ELISA was adapted, which allows testing drug covering a broad range of concentrations and rapid spectrophotometric analysis using an automated plate reader. An *in situ* ELISA method was described by Figueiredo & Shope (1987) [31], and then used with the objective of diagnosis, surveillance and serum-epidemiological studies for the detection of IgM and IgG antibodies using infected mosquito cells as antigen [32]. However there are few reports using the *in situ* ELISA as an anti-dengue drug screening technique. Ying Wang *et al*. (2009) [12] named an *in situ* ELISA as cell-based *Flavivirus* immunodetection (CFI) assay, and it was used to screen the anti-dengue virus replication activity of 111 compounds in A549 cell line. The same assay was later used to assess the inhibition of DENV RNA synthesis by an adenosine nucleoside [33] and an ester prodrug of an adenosine analog [34].

To obtain better results, several aspects of the assay were optimized. Initially, fixative reagents were evaluated. The cell fixation procedure using the organic solvents methanol and acetone showed better results when compared to fixation with paraformaldehyde and permeabilization with triton X-100 (Figure 2A). Another advantage of the first fixation procedure is that it precipitates proteins and dissolves lipids from cell membranes making them permeable to antibodies at the same time [35]. Additionally, blocking unspecific reaction using 2% skim milk or 5% FBS demonstrated similar efficiency, and 2% skim milk was selected for its availability.

Next, the cell density per well was evaluated, and six different concentrations of Huh7.5 cells in the range of 5×10^3^ and 1.6×10^5^ per well (two fold dilution) were seeded. After 16 h, cells were infected with DENV-1/FGA/89 with a multiplicity of infection (MOI) of 4 for 1 h30 min, then the inoculum was removed, complete medium was added and plates were incubated for additional 72 h. The intermediate concentration tested (8×10^4^–2×10^4^) yielded the highest absorbance readings (Figure 2B) and the cell density of 2×10^4^ showed the best signal to background rate (S/B = 5.8) and was used for further tests. Cells at a concentration higher than 1.6×10^5^ per well tended to overgrow and detach from the wells during washes, giving lower absorbance and higher background values.

The final steps for the *in situ* ELISA standardization were to define the MOI for each DENV serotype and the time of incubation after infection. For all DENV serotypes, with exception of DENV-4/TVP360, the highest absorbance values were observed with a MOI of 4 at 72 hours post infection. DENV-4/TVP360 presented high optical density (OD) readings for both time points and even for the lowest MOIs tested, then the MOI of 0.1 at 72 h post infection was chosen (Figure 2C and 2D); this might be related to the fact that this is a laboratory strain adapted to cell culture. In relation to time of incubation after the infection, a longer period of incubation as expected showed higher OD values, probably because it allowed for more cycles of virus replication.

The *in situ* ELISA assay described here constitutes a rapid and reliable screening method for inhibitors of DENV infection in mammalian cells, in which the DENV envelope (E) protein was detected using the 4G2 antibody. Previous studies have confirmed that the level of E protein is reflective of the level of infectious virus production and that a reduced production of E protein could potentially be an indication of reduced viral entry, genome replication, or protein translation and processing [19].

### Antiviral Assay Validation

To validate the proposed *in situ* ELISA, it was compared to the foci-forming assay commonly used to detect virus infection inhibition [14], [24]. Both assays were performed in parallel using cells and supernatant from the same culture. The comparison between OD values and virus titer for DENV-1, -2 and -3 yielded linear and highly correlated data (average r^2^ = 0.95). However this result was not observed for DENV-4 (Figure 3A), as it shows high OD and titer values for all the MOIs tested, maybe because DENV-4/TVP360 is a laboratory strain adapted to cell culture, different from the other low passage clinical isolates used in this study. In addition the comparison of the dose response curve (DRC) and the IC50 for IFN-α 2A and DENV-4 infection by the foci-forming assay and a commercial NS1 antigen capture ELISA assay (Figure 3B), confirmed that the *in situ* ELISA is reliable to screen antiviral activities.

IFN-α is an antiviral drug used in the treatment of hepatitis C infection [36], and proved to be an effective *in vitro* replication inhibitor of several pathogenic flaviviruses, including dengue [37]. Based on that, interferon-α 2A was used as an reference control, and showed a dose dependent virus inhibition with a mean IC50 of 2.94; 2.32; 2.64 and 5.70 IU/ml for DENV serotypes 1, 2, 3 and 4 respectively (Figure S1). The concentration of 100 IU/ml was used as the IC90 and the positive control of the assay.

### Seaweed Extracts Cytotoxicity

A disadvantage of the *in situ* ELISA assay for the screening of antiviral agents is that it does not give information about compound cytotoxicity, thus prior to antiviral testing it is important to apply a cell viability test. Several reports show that some seaweed extracts or compound isolated from seaweeds have cytotoxic effect in cancer cells [38], [39]. To ascertain the seaweed extracts cytotoxicity in Huh7.5 cells we choose to use two assays that assess cell viability through different mechanisms. The MTT assay, which is based on the reduction of the yellow tetrazolium salt MTT to purple formazan crystals by living cells with active cellular reductases in the mitochondria [40] and the N-red uptake assay that is based on cell’s capacity to maintain pH gradients in lysosomes, through the production of ATP [41]. A dose response curve was obtained for each seaweed extract, the CC50 was calculated and the maximum non-toxic dose (MNTD) was determined for the antiviral test based on both assays (Table 1). We found similar results for CC50 between the two assays, and N-red seems to be more sensitive than the MTT assay, corroborating with a previous study that shows that the N-red presents less interference, is more sensitive and does not use unstable reagents as required for the viability tests using tetrazolium salts (MTT, MTS, XTS) [42]. Seaweed extract A7 was highly toxic and was not tested in the screening assay.

### Seaweed Extracts Antiviral Screening

Natural products offer a privileged starting point in the search for specific and potent modulators of biomolecular function as well as novel drugs [43]. The marine environment is of great importance to the global biodiversity. It is predicted that there are ∼8.7 million eukaryotic species globally, of which ∼2.2 million are marine, offering an almost infinite resource for novel compounds [44], [45].

Marine organisms are known producers of pharmacological and antiviral agents and may provide unlimited biological resources for the production of therapeutic drugs against viral infections in humans. Extracted compounds from seaweeds have *in vitro* and/or *in vivo* activity against a wide range of viruses, including herpes viruses (HSV-1, HSV-2, HCMV), togaviruses (Sindbis virus, Semliki Forest virus), paramyxoviruses (RSV), rhabdoviruses (VSV), and both human and simian immune deficiency viruses (HIV and SIV) [17]. In relation to dengue virus, a sulfated polysaccharide named fucoidan showed a potent inhibition when DENV-2 was pretreated with it, however none effect was observed for other DENV serotypes [46].

The seaweed extract antiviral screening against all dengue serotypes was performed in the 96-well format *in situ* ELISA. The well-to-well variation was evaluated by the measurement of standard statistical parameters, the average S/B was 7.2 and Z-factor was 0.62. Fifteen seaweed extracts were screened at the MNTD, and extracts A1, A2, A3, A8, A9, A11, A12 and A14 presented a statistical dengue infection inhibition when compared to controls (Figure 4).

A study with two sulfated polysaccharides obtained from the red seaweeds *Gymnogongrus griffithsiae* and *Cryptonemia crenulata* showed that the antiviral activity against dengue was dependent on virus serotype and host cell. It was demonstrated a considerable inhibition of DENV-2 multiplication in Vero cells, lower antiviral effect against DENV-3 and DENV-4, and no effect against DENV-1 [27]. In our study we also observed such differences and in order to ascertain if it was serotype or strain specific, four seaweed extracts were chosen among the hits for DENV-1 and -3, being two from the *Phaeophyta* (A1 and A3), one from the *Rhodophyta* (A8) and one from the *Chlorophyta Phylum* (A12). These extracts were tested against other strains from each serotype, except DENV-4 for its unavailability (Figure 5). The results demonstrate that the infection inhibition by these extracts seems to be serotype and strain independent.

### Seaweed Extracts A1, A3, A8 and A12 Act at an Early Stage during Dengue Virus Infection

All the experiments up to here were performed as post-infection treatment, in this way we might have missed compounds with mechanism of action in early infection steps, like virus adsorption and entry or even virucidal effect. Harden *et al.* (2009) [47] evaluated the antiviral activity of extracts from *Undaria pinnatifida*, *Splachnidium rugosum*, *Gigartina atropurpurea*, and *Plocamium cartilagineum* against HSV-1 and HSV-2. These extracts exhibited good activity when added during the first hour of viral infection, but were ineffective if added later and subsequent assays showed that the compounds had potent virucidal activity and were active at very low concentrations.

Time of addition experiments (Figure 6A) with the previously chosen extracts and DENV-4 infection pointed that these seaweed extracts might act at an early stage of the virus infection cycle. These results were confirmed by the foci-forming assay (Figure 6B).

Considering that the treatment during the infection (time 0 h) showed a high inhibition rate, DRC was performed using this treatment and the IC50 and SI were obtained (Figure 7). These four extracts showed promising SI values, and were comparable to glycyrrhizin anti-DENV treatment in Vero cells [37].

A possible virucidal effect of the seaweed extracts A3, A8 and A12 was discarded by the virucidal assay (Figure S2), and A1 might have a partial virucidal activity. These extracts were further evaluated in binding and internalization assays (Figure 8). Taken together, the results show that the major inhibition occurs during the binding process for seaweed extracts A1 and A3, with a lower inhibition during internalization. For the extracts A8 and A12 it seems that both steps are being inhibited.

In regard to the seaweed extracts composition, a previous study with similar dichloromethane/methanol extraction of two populations of *Canistrocarpus cervicornis* (source of A1) presented a total of fourteen diterpenes, among them dolastanes, ecodolastanes and seco-dolastane were detected. The results revealed a typical pattern for *C. cervicornis*, which did not vary according to the geographical region [25]. From *Padina gymnospora* (source of A3) only few metabolites classes were isolated until now, such as fatty acids [48], and sulfated polysaccharides [49]. From *Palisada perforada* (source of A8) only two triquianes alcohols compounds were described [50]. And from genus *Caulerpa* (source of A12) was previously isolated fatty acid, sterols, terpenes, and alkaloids [51].

### Conclusions

Data from our study demonstrated the standardization and validation of an *in situ* ELISA assay in a human cell line (Huh7.5) for the screening of antiviral compounds against all DENV-serotypes. The assay constitutes a reliable screening method for inhibitors of DENV infection in mammalian cells and shows to be reproducible with other dengue virus quantification techniques like the traditional foci-forming assay and the NS1 antigen capture ELISA. This standardized technique can be automated making possible the screening of larger number of compounds in a short period of time and with lower costs than other high-throughput methods. Additionally, the technique proved to be useful in the screening of 15 seaweed extracts isolated from the Brazilian coast, and demonstrated that 8 seaweed extracts were able to reduce DENV-antigen production in Huh7.5. Further studies demonstrated that the seaweed extracts A3, A8 and A12 are not virucidal, but seems to act at an early stage of the virus infection cycle, as shown by the time of addition experiment, and that the antiviral activity is serotype independent. There is an important inhibition of the binding process by A1 and A3, with lower suppression during internalization, and it seems that both steps are inhibited by A8 and A12 seaweed extracts. These extracts also presented a good selectivity index and will be further analyzed regarding the extract composition.

## Supporting Information

Figure S1 **Dose response curve for interferon-α 2A.** **(A)** Cells were seeded and infected with DENV-1, **(B)** DENV-2 and **(C)** DENV-3 with an MOI of 4, and **(D)** DENV-4 with an MOI of 0.1. After 1 h30 min the inoculum was removed and the culture was treated with IFN-α 2A at a range of concentrations (1000–0.08 IU/ml). Values represent mean ± SD of three independent experiments and were analyzed by sigmoidal dose-response curve (variable slope).
(TIF)

Figure S2 **Virucidal Assay.** DENV-4 was incubated with the seaweed extracts A1, A3, A8 and A12 at the MNTD, in the presence or absence of RNase. After 1 h incubation at 37°C, RNA was isolated and subjected to RT-PCR and gel electrophoresis.
(TIF)
