# Supplementary figures and images for: Screening of Dengue Virus Antiviral Activity of Marine Seaweeds by an In Situ Enzyme-Linked Immunosorbent Assay
Source: PLoS One. 2012 Dec 5;7(12):e51089. doi: 10.1371/journal.pone.0051089 (PMC3515490; doi:10.1371/journal.pone.0051089)

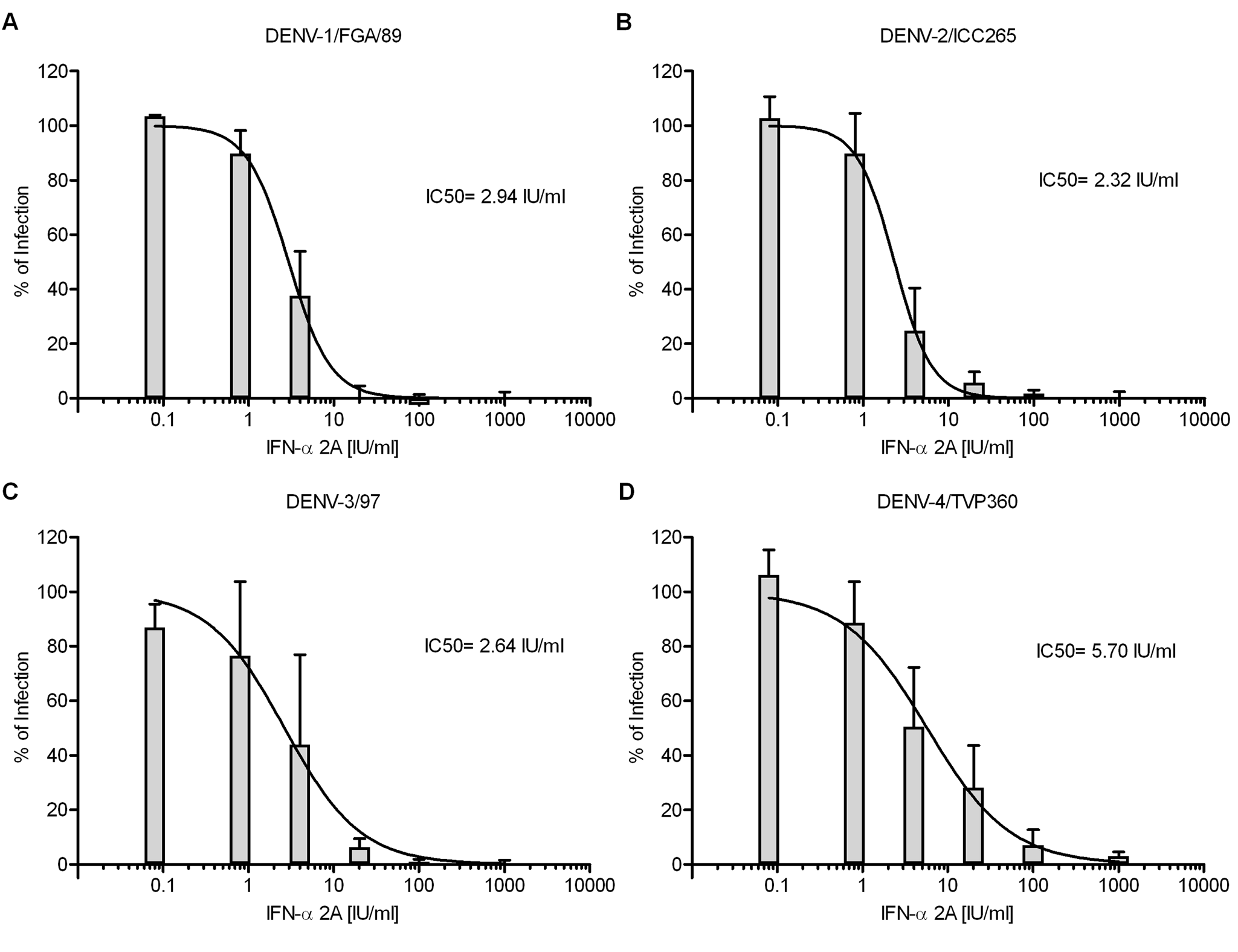

Supplement: Figure S1 — Dose response curve for interferon-α 2A. (A) Cells were seeded and infected with DENV-1, (B) DENV-2 and (C) DENV-3 with an MOI of 4, and (D) DENV-4 with an MOI of 0.1. After 1 h30 min the inoculum was removed and the culture was treated with IFN-α 2A at a range of concentrations (1000–0.08 IU/ml). Values represent mean ± SD of three independent experiments and were analyzed by sigmoidal dose-response curve (variable slope). (TIF) [file pone.0051089.s001.tif]
